# Veterinary Medical Association, American Veterinary College

**Published:** 1895-03

**Authors:** 


### VETERINARY MEDICAL ASSOCIATION, AMERICAN VETERINARY COLLEGE.

The Medical Association of the American Veterinary College has been holding weekly meetings during the present session, and a great deal of interest has been awakened among the students in this line of work. The officers for the present year are: President, W. J. Coates; Vice-President, A. Blake; Secretary, Frank Hanson; Treasurer, A. Mitchell.

The following papers have been read and discussed, and a great deal of interest aroused: "Navicular Disease and Neurotomy," "The Microscope as a Means of Diagnosis," "Lymphangitis," "Purpura Hemorrhagica," "Electricity in Medicine and Surgery," The Thermometer as an Aid to Prognosis," "Tetanus," "Azoturea," "Modes of Administering Medicine," "Milk Inspection," "Diseases of the Foot," "Diseases of the Hock," "Hog Cholera," "Pathological Horse-shoeing," "Bath in Disease."

The meetings are held on Friday in the College hall.

